# Comparison of two nutrition assessment tools in surgical elderly inpatients in Northern China

**DOI:** 10.1186/s12937-015-0054-8

**Published:** 2015-07-14

**Authors:** JunDe Zhou, Miao Wang, HaiKuan Wang, Qiang Chi

**Affiliations:** 1 Department of General Surgery, The Second Affiliated Hospital, Harbin Medical University, 148, Bao Jian Road, Harbin, 150081 China; 2 Center for Animal Disease Control of Heilongjiang Province, Harbin, 150069 China

**Keywords:** Malnutrition, Nutritional assessment, Short-form mini-nutritional assessment, NRS2002

## Abstract

**Background & Objective:**

Nutrition assessment enables early identification of malnourished patients and those at risk of malnutrition. To determine the prevalence of malnutrition, to analyze the correlation between short-form Mini Nutritional Assessment (MNA-SF) and Nutritional Risk Screening 2002 (NRS2002) with classical nutritional markers among elderly hospitalized patients in surgery departments, with a view to improving nutrition advice for these patients.

**Methods:**

A total of 142 elderly patients admitted for surgery were enrolled in the study. Within 48 hours of admission, MNA-SF and NRS2002 scale, anthropometric measures and biochemical tests were carried out to assess the nutritional status of each patient.

**Results:**

The prevalence of malnutrition classified by MNA-SF, NRS2002, BMI, serum albumin, hemoglobin, total lymphocyte count, handgrip strength, calf circumference and mid-arm circumference were 45 %, 38 %, 17 %, 22 %, 24 %, 71 %, 36 %, 12 % and 15 %, respectively. As the nutritional status classified by both MNA-SF and NRS2002 deteriorated, BMI, serum albumin, hemoglobin, handgrip strength, mid-arm circumference and calf circumference of patients with malnutrition were lower (*P* < 0.05). MNA-SF and NRS2002 had a unanimous correlation with classical nutritional markers (*P* < 0.05) except total lymphocyte count (*P* > 0.05). MNA-SF results showed a moderate agreement (*P* < 0.001) with NRS2002. Malnourished patients were older than well-nourished patients with NRS2002 (*P* < 0.05). Digestive disease patients tend to suffer from malnutrition, evaluated by MNA-SF (*P* < 0.05).

**Conclusions:**

The results show a relatively high prevalence of malnutrition among elderly patients in our general surgery department, especially in patients with digestive disease. NRS2002 and MNA-SF on elderly patients showed great consistency but significant difference in elderly patients with digestive disease. Both MNA-SF and NRS2002 correlated with each other and with BMI, serum albumin, hemoglobin, handgrip strength, calf circumference and mid-arm circumference. MNA-SF may be a more suitable tool for the nutrition assessment of surgical elderly inpatients.

## Introduction

The negative health consequences of malnutrition in elderly hospitalized patients have been extensively documented, and it is also well known that malnutrition is an under-recognized and undertreated problem throughout the healthcare system. Clinically, hospital malnutrition may contribute to an increase in the number and severity of disease complications by increasing morbidity and mortality [1]. The identification and treatment of malnutrition earlier can lead to improved outcomes and better quality of life. Therefore, the development of appropriate tools to assess the degree of malnutrition in patients is essential. Malnutrition assessment has been recommended to identify accurately those individuals who have clinically significant malnutrition by the American Society for Parenteral and Enteral Nutrition (ASPEN), the European Society for Clinical Nutrition and Metabolism (ESPEN), Japanese Society for Parenteral and Enteral Nutrition (JSPEN) and the Chinese Society for Parenteral and Enteral Nutrition (CSPEN) [2, 3].

As there is no ‘gold standard’ for the assessment of nutritional status in hospitalized patients, a variety of assessment methods and indicators (biochemical tests and anthropometric indexes), which are epidemiologically related to patients’ morbidity and mortality, have been reported in the literature either alone or in combination to diagnose malnutrition both nationally and internationally; however, they have limitations when considered alone. The use of single objective nutrition parameters to assess nutritional status has been questioned due to the low predictive value and lack of sensitivity and specificity, as many non-nutritional factors affect the results. Body mass index (BMI) has traditionally been used, but short-form mini nutritional assessment (MNA-SF) and Nutritional Risk Screening 2002 (NRS2002) have also been used more recently. The MNA-SF and NRS2002 are valid methods for nutrition assessment of malnutrition in the elderly (>65 years) both in the community and hospital.

In this paper, we sought to investigate the frequency of malnutrition in elderly inpatients (≥65 years), hospitalized in the surgery department of the Second Affiliated Hospital, Harbin Medical University in China. The nutritional status of 142 hospitalized patients admitted to the surgery ward, were evaluated by MNA-SF and NRS2002, routine anthropometric measurements and laboratory tests undertaken, and the data analyzed. Collectively, this analysis will help us design complementary studies and redefine preventive plans and treatment regimes for malnutrition.

## Materials and methods

### Ethics

This study was approved by the relevant research ethics committee in Harbin, China. All the patients recruited and/or their next of kin were informed about the study before participating and signed written consent forms, before the interview and assessment of their nutritional status. Ethics guidelines and subjects’ confidentiality were strictly followed throughout the study. As part of the ethical screening practice, patients identified as being at nutritional risk by either method were referred to their medical doctors.

### Patients

We conducted an observational, cross-sectional and descriptive study. Fixed-point consecutive sampling was adopted in the surgery department of the Second Affiliated Hospital, Harbin Medical University (China) between February 2012 and January 2013. Therefore, a total of 142 patients (76 males) were finally studied, including 104 with digestive system disease and 38 non-digestive system disease patients. All patients were over the age of 65 years, the median age of which was 71.9 years old (range, 65–85 years).

**Eligibility criteria included:** (a) Patients ≥ 65 years old. (b) Patients scheduled for surgery. (c) A stay > 24 h in hospital. (d) Patients had given informed consent.

**Exclusion criteria included:** (a) Patients with cognitive impairment, known mental disorder or who were comatose. (b) Patients with communication problems (c) Previous surgery, chemo/radiotherapy during the year prior to hospital admission (d) Patients unable to perform laboratory tests or anthropometric measurements. (e) Patients with critical illness, acute disease or infection, needing treatment prior to nutritional assessment at the time of admission. (f) Patients with diabetes, severe liver or renal dysfunction. (g) Hyponatremia (≤135 mmol/L) and hypernatremia (≥145 mmol/L) due to interaction with serum albumin. (h) Less than 65 years old. (i) Patients lost during follow up or with incomplete data.

### Data collection

Due to the lack of a universally accepted ‘gold standard’ for grading the nutritional status of the recruited patients, we used a batch of indicators of clinical relevance as external standards. All subjects underwent the collection of personal characteristics, anthropometric measurements and laboratory tests within the first 48 h after presentation. Personal characteristics about gender, age, race/ethnicity, primary diagnosis and co-existing comorbidities, date of admission and hospital discharge were collected at baseline from clinical files, directly by patients when this information was not available in their files. Anthropometric parameters and laboratory tests are presented as follows.

#### Anthropometric parameters

Anthropometric measurements were taken following the standard procedures described by Lohman and colleagues [4]. Weight, height, BMI, handgrip strength (HGS), mid-arm circumference (MAC), and calf circumference (CC) were performed as a part of anthropometric measures following established procedures [5, 6].

Current weight (kg) and height (m) were measured using calibrated scales with a stadiometer (RGZ-120 weight/height scale, China). Body weight was measured to the nearest 0.2 kg with light ward uniform and without shoes, in fasted patients. Height was measured to the nearest 0.5 cm, without shoes. BMI (kg/m^2^) was calculated (body weight (kg)/(height in meters)^2^ as proposed by Campillo et al. [6]. Nutrition status was defined as nutritional deficiency if BMI < 20.5 kg/m^2^, well-nourished if BMI ≥ 20.5 kg/m^2^ according to the Chinese Chen Chunming standard for BMI assessment [7]. Percentage of unintentional weight loss over the last 3 and 6 months was recorded following patients’ reports.

HGS, reflecting early changes in muscle function and correlating well with nutritional status, was measured in the early morning to the nearest 0.5 kg using a mechanical handgrip dynamometer. Three measurements were taken and the highest was recorded. The patients were classified as malnourished when their HGS was below the tenth percentile [8].

MAC and CC were measured following standard procedures described by Lee et al. [9], and all measurements were taken in duplicate and accurate to 0.1 cm. Additional measurements above and below the point were made to ensure that the first value was the largest. MAC and CC classification were performed by using the percentage of adequacy through the method proposed by Blackburn and Harvey [10], and patients with percentage of adequacy < 90 % were considered as under-nourished. Measured values of MAC and CC were divided by respective cut points values (MAC = 22.5/21 cm and CC = 28/25 cm for males/females, respectively) for standardization [11].

#### Laboratory tests

The following laboratory tests were carried out using standard methods: hemoglobin (Hb), total lymphocyte count (TLC), and albumin (Alb), close to the day on which the anthropometric indexes, NRS2002 and MNA-SF were carried out. Blood samples drawn from all patients on admission were analyzed in the central lab of the Second Affiliated Hospital, Harbin Medical University. The cutoff value for Alb measured by immunonephelometry was set at 35 g/L (normal range 35–55 g/L) as an indicator of under-nourished [12]. The cutoff value for TLC was < 2.0 × 10^3^/mm^3^ for both genders, for depletion diagnosis as proposed by Blackburn et al. [13]. Hb was compared with reference values for males (120 g/L) and females (110 g/L), respectively.

### Nutrition screening and assessment

All recruited patients underwent the following two types of nutritional evaluation (MNA-SF and NRS2002) and the results are presented in Table 1. These tools are often utilized in clinical practice and clinical research efforts, to assess the nutritional status of patients within 48 h of hospital admission. After assessment, all study participants were followed up throughout their hospital stay until discharge or death.

**Table 1:** Malnutrition screening tools for elderly hospitalized populations [11]

Tools	Year of validation	Parameters	Initial purpose	Cutoff score
MNA-SF [2]	2001	weight change, recent intake, BMI, acute disease, mobility, dementia/depression,	To detect malnutrition in the elderly	12-14 normal nutritional status ≤ 11 under-nourished
NRS2002 [3–5]	2002	weight loss history, recent intake, BMI, severity of disease, age	To detect malnutrition and identify patients who need closer monitoring	0-2 well nourished ≥ 3 under-nourished

*MNA-SF* Mini Nutritional Assessment Screening Form, *NRS2002* Nutritional Risk Screening 2002

#### NRS2002

NRS2002 was developed by the Danish Association of Parenteral and Enteral Nutrition (DAPEN), and was recommended by ESPEN. NRS2002 was designed as a tool to identify patients at nutritional risk and is a valid and reliable tool for assessing the nutritional status of elderly hospitalized patients. The NRS2002 structured nutritional evaluation test was administered to patients for whom laboratory studies were ordered on admission, according to the recommendations of Kondrup and colleagues [2, 14, 15].

Nutritional risk was assessed through two criteria namely impaired nutritional status and disease severity. A score between 0 and 3 was given according to the recommendations for each criteria. Nutritional status was determined by three variables: BMI, recent weight loss, and food intake during the week before admission. Disease was analyzed as an indicator of metabolic stress and increased nutritional requirements. For people aged ≥ 70 years, an additional score was awarded (age adjustment). The NRS2002 score is the total of the nutritional score, severity of disease score and the age adjustment score. Patients with a total score of ≥ 3 were considered as under-nourished, and indicated that nutrition support should be initiated. Patients were classified as at no risk (≥3) or under-nourished (at nutritional risk/malnourished) [16].

#### MNA-SF

The MNA-SF, revised screening form of Mini-Nutritional Assessment (MNA) was developed especially for older (>60 years) patients. It relies on 6 questions (appetite, weight loss, mobility, recent illness/stress, dementia/depression and BMI), and is scored from 0 to 3. A normal nutritional status was denoted by a score >11 points (12 ~ 14), under-nourished (at nutritional risk/malnourished) if the score was 11 or less. For those participants unable to stand independently, we used the CC to substitute BMI as proposed by Kaiser MJ [17] and (http://www.mna-elderly.com).

### Quality control

We defined older patients as those ≥ 65 years of age. None of these patients was receiving nutritional support at the time of assessment. All interviews, measurements and data collection were performed during the patients’ preoperative period in a single session, by the same trained researcher who performed all of the nutritional status assessments (NRS2002 and MNA-SF). A standardized nutritional assessment questionnaire was used for screening and assessment. Moreover, the researcher was not aware of the laboratory test results at the time of the assessment. The predictive value of each scale was evaluated by comparing the ability to differentiate under-nourished based on a batch of biochemical and anthropometric measurements (as external standards). Additionally, a comparison study was conducted according to the tables proposed by Barbosa-Silva et al. [18]. The values of NRS2002 and MNA-SF were considered to be reduced when the results were lower than the above criteria (Tables 1). All medical records were retrieved and examined by the first author. Moreover, the attending doctor was informed if a patient was regarded as under-nourished using these methods.

### Statistical analysis

Statistical analyses were completed using SPSS version 16.0 for Windows (SPSS Inc. , Chicago, IL, USA). The nutritional indicators were dichotomized into under-nourished and without malnutrition as proposed in the literature [16, 19, 20]. Quantitative data were expressed as the mean ± standard deviation, and qualitative data were expressed as percentages. Differences in mean values were tested with one-way analysis of variance and Student’s *t*-test for normal data. Differences in qualitative data were assessed using a chi-square test. Spearman’s correlation was carried out to show the correlation between NRS2002, MNA-SF and other nutritional parameters. A concordance analysis using the kappa coefficient was calculated to measure the rate of agreement between the two methods. The results were interpreted as follows: ≤ 0.20, poor agreement; 0.21 to 0.40, weak agreement; 0.41 to 0.60, moderate agreement; 0.61 to 0.80, substantial agreement; and 0.81 to 1.00, almost perfect agreement [21, 22]. To compare the accuracy of each screening tool to detect malnutrition, the sensitivity, specificity, positive predictive value (PPV) and negative predictive value (NPV) were calculated. Statistical significance was set at *P* < 0.05 for all tests.

## Results

Over the 12-month study period, a total of 142 individuals, including 104 with digestive system disease and 38 with non-digestive system disease from 10 surgery wards of the Second Affiliated Hospital, Harbin Medical University, China, met the eligibility criteria and completed a nutrition assessment within 48 h of admission. All patients were ≥ 65 years old, the average age being 71.8 ± 5.4 years for women (range, 65–82 years) and 72.0 ± 5.9 years for men (range, 65–85 years).

The baseline characteristics of these patients are summarized in Tables 2 and 3. Table 2 shows malnutrition prevalence, according to the different methods adopted in our study. According to MNA-SF, 55 % of the sample patients were considered to be well nourished, and 45 % under-nourished. Based on the results of the internationally validated NRS2002, 62 % of patients were considered to be well nourished, and 38 % at risk of malnutrition. According to the criteria defined above, the prevalence of undernutrition varied from 12 to over 71 % depending on the tool used. The classifications by MNA-SF, NRS2002, BMI, Alb, Hb, TLC, HGS, MAC and CC were 45 %, 38 %, 17 %, 22 %, 24 %, 71 %, 36 %, 15 % and 12 %, respectively.

**Table 2:** Nutritional status (*n*, %) of 142 patients classified with the MNA-SF A, NRS2002, serum and anthropometric parameters

	Undernutr.	Normal
NRS2002	54 (0.38)	88 (0.62)
MNA-SF	64 (0.45)	78 (0.55)
BMI	24 (0.17)	118 (0.83)
Alb	31 (0.22)	111 (0.78)
Hb	34 (0.24)	108 (0.76)
TLC	101 (0.71)	41 (0.29)
HGS	51 (0.36)	91 (0.64)
CC	17 (0.12)	125 (0.88)
MAC	22 (0.15)	120 (0.85)

*MNA-SF* revised screening form of Mini-Nutritional Assessment, *NRS2002* Nutritional Risk Screening 2002, *Undernutr* under-nourished (malnourished + at risk of malnutrition), *Alb* serum albumin, *TLC* total lymphocyte count, *HGS* handgrip strength, *MAC* mid-arm circumference, *CC* calf circumference

**Table 3:** Anthropometric and biochemical characteristics of subjects

	NRS			MNA-SF		
	Normal	Undernutr.	P	Normal	Undernutr.	P
	(*n* = 88)	(*n* = 54)		(*n* = 78)	(*n* = 64)	
Age	70.86 ± 5.72	73.57 ± 5.18	0.005	71.50 ± 5.64	72.38 ± 5.68	0.358
BMI	25.22 ± 3.28	21.64 ± 2.96	<0.0001	25.32 ± 3.20	22.08 ± 3.26	<0.0001
Alb	41.11 ± 6.73	38.21 ± 6.34	0.01	41.82 ± 6.47	37.81 ± 6.38	0.0003
Hb	132.93 ± 16.66	121.39 ± 23.57	0.0008	133.86 ± 17.98	122.06 ± 21.18	0.0004
TLC	1.71 ± 0.77	1.70 ± 0.67	0.94	1.74 ± 0.71	1.66 ± 0.76	0.52
HGS	24.40 ± 19.32	18.01 ± 15.54	0.04	24.91 ± 19.52	18.38 ± 15.85	0.03
CC	31.28 ± 3.09	29.63 ± 3.24	0.003	31.59 ± 3.11	29.51 ± 3.04	0.0001
MAC	25.39 ± 2.41	23.09 ± 2.68	<0.0001	25.39 ± 2.42	23.46 ± 2.77	0.00002

*MNA-SF* revised screening form of Mini-Nutritional Assessment, *NRS2002* Nutritional Risk Screening 2002, *Undernutr* under-nourished (malnourished + at risk of malnutrition), *Alb* serum albumin, *TLC* total lymphocyte count, *HGS* handgrip strength, *MAC* mid-arm circumference, *CC* calf circumference

Table 3 shows that BMI, MAC, CC, HGS, Hb and Alb differed between malnourished and well-nourished groups according to both assessments. TLC did not differ between groups with either assessment. Malnourished patients had lower levels of BMI, MAC, CC,, HGS, Hb and Alb (*P* < 0.05). Under-nourished patients were older than well-nourished patients with NRS2002 (*P* < 0.05). We noticed some differences but age and MNA-S did not achieve statistical significance (*P* > 0.05).

**Fig. 1:**
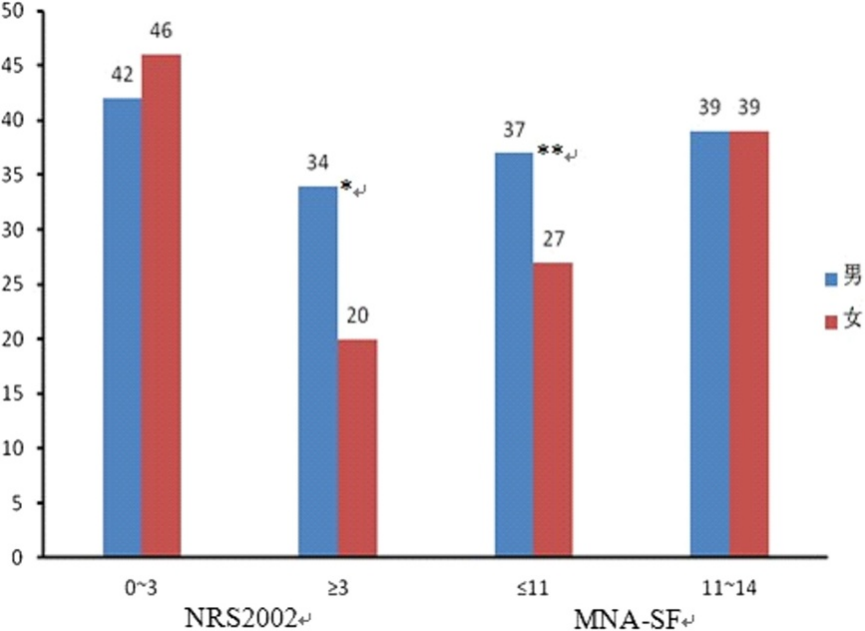
Malnutrition prevalence according to MNA-SF and the NRS2002, in the total sample and by gender. Abbreviations: MNA-SF, short form of Mini-Nutritional Assessment. NRS2002, Nutritional Risk Screening 2002. Under-nourished was defined as NRS2002 ≥ 3 and MNA-SF ≤ 11. *&** *P* > 0.05 (between gender)

Nutritional status classified with MNA-SF and NRS2002, and stratified by gender, showed different results (Fig. 1). Among those patients detected under-nourished, only 26.06 % was male according to the MNA-SF (*P* > 0.05). Among those patients detected under-nourished, only 23.94 % was male, while 14.08 % was female, according to the NRS2002 (*P* > 0.05).

Table 4 reveals that MNA-SF showed a moderately low consistency (Kappa = 0.5961, *P* < 0.001) with NRS2002.

**Table 4:** Kappa test for agreement in diagnosing malnutrition between NRS2002 and MNA-SF scores (*n* = 142)

	MNA-SF		
	Undernutr.	Normal	Total
NRS2002			
Undernutr.	45 (0.83)	9 (0.17)	54
Normal	19 (0.22)	69 (0.78)	88
Total	64	78	142
Kappa (95 % CI)	0.5961 (0.4637,0.7285)		
*P*	<0.0001		

*MNA-SF* short form of Mini-Nutritional Assessmen, *NRS2002* Nutritional Risk Screening 2002. *Undernutr* under-nourished (malnourished + at risk of malnutrition)

Table 5 shows the Spearman correlation coefficients of MNA-SF and NRS2002 scores according to age, BMI, serum and anthropometric parameters. BMI, serum and anthropometric parameters correlated positively with malnutrition scores of MNA-SF, and correlated inversely with the scores of NRS2002 (*P* < 0.05). Contrary to BMI, serum and anthropometric parameters, age correlated inversely with MNA-SF and NRS2002. TLC had no correlation with MNA-SF and NRS2002 scores (*P* > 0.05)

**Table 5:** Spearman correlation coefficients of MNA-SF and NRS2002 scores with serum and anthropometric parameters

	NRS2002		MNA-SF	
	rs	*P*	rs	*P*
Age	0.33	<0.0001	−0.11	0.2137
BMI	−0.43	<0.0001	0.56	<0.0001
Alb	−0.326	<0.0001	0.28	0.0006
Hb	−0.331	<0.0001	0.31	0.0002
TLC	−0.11	0.1903	0.12	0.1494
HGS	−0.21	0.0126	0.17	0.0484
CC	−0.25	0.0023	0.38	<0.0001
MAC	−0.398	<0.0001	0.43	<0.0001

The total score of MNA-SF and NRS2002 did not contain the score contributed by the respective anthropometric parameters and serum parameters when calculating the correlation of the MNA-SF and NRS2002 scores with each of these specific parameters

*MNA-SF* revised screening form of Mini-Nutritional Assessment, *NRS2002* Nutritional Risk Screening 2002, *Undernutr* under-nourished (malnourished + at risk of malnutrition), *Alb* serum albumin, *TLC* total lymphocyte count, *HGS* handgrip strength, *MAC* mid-arm circumference, *CC* calf circumference

Table 6 shows that digestive system disease patients tended to suffer from malnutrition, evaluated by MNA-SF (*P* < 0.05), while there is no statistical significance with NRS2002, depending on the admission department (*P* > 0.05).

**Table 6:** Nutritional status of 142 patients of different etiology classified with the MNA-SF A and the NRS2002

	NRS2002				MNA-SF			
	Normal	undernutr.	χ2	*P*	Normal	undernutr.	χ2	P
	(*n* = 88)	(*n* = 54)			(*n* = 78)	(*n* = 64)		
Digestive disease	60	44	3.02	0.08	48	56	12.09	0.0005
Non-digestive disease	28	10			30	8		

*MNA-SF* revised screening form of Mini-Nutritional Assessment, *NRS2002* Nutritional Risk Screening 2002, *Undernutr* under-nourished (malnourished + at risk of malnutrition)

The assessment of nutritional status according to the Alb was performed in all patients. The sensitivity, specificity, and positive and negative predictive values of the studied nutritional screening tools with respect to the assessment by Alb are presented in Table 7. Specificity with NRS-2002 was high (65.77 %), but it showed lower sensitivity (51.61 %). MNA-SF had a poor specificity (61.26 %) and a high sensitivity (67.74 %).

**Table 7:** Sensitivity, specificity, Youden index and positive and negative predictive values of nutritional screening tools to determine malnutrition by the Alb when used in 142 patients

	Sensitivity (%)	Specificity (%)	PPV (%)	NPV (%)	Youden index (%)
NRS2002	51.61	65.77	29.63	82.95	17.38
MNA-SF	67.74	61.26	32.81	87.18	29

Sensitivity and specificity results expressed as percentage (95 % CI)

*MNA-SF* short form of Mini-Nutritional Assessment; *NRS2002* Nutritional Risk Screening 2002, *PPV* Positive predictive value, *NPV* Negative predictive value, *NRS-2002* Nutritional Risk Screening 2002

## Discussion

Malnutrition in hospitalized patients is a critical issue and is associated with poor wound healing, higher post-operative infection risk, adverse functioning of the gastrointestinal tract, increased morbidity and mortality, decreased treatment efficacy, and an increased hospital stay period [23, 24]. Worldwide studies have indicated that between 20 and 50 % of hospitalized patients have some degree of malnutrition. Despite the greater awareness of this condition by healthcare staff and improvements in the assessment of malnutrition, multiple reports have indicated that only a minority of malnourished patients actually receives appropriate nutrition support while hospitalized [25, 26]. Nutritional screening should be the first step to identify patients who are malnourished or are at risk of malnutrition, for early referral, further nutritional assessment and individualized intervention [27]. Several conventional approaches and criteria such as BMI, biochemical markers and anthropometric measurements could be used alone or in combination to diagnose malnutrition. In the clinical setting, most of the anthropometric measurements and laboratory assessments are not ideal because they are inaccurate, insensitive or unconvenient to perform.

Apart from classical methods, composite nutrition assessment tools should be used for nutritional assessment, such as the MNA-SF and NRS2002. In this study, the nutritional diagnosis of elderly surgical inpatients were assessed by using MNA-SF, NRS2002, BMI, HGS, AC, CC, Alb, Hb and TLC. These methods and criteria were chosen because they are available, they have fast application and low cost, and they can be incorporated into the routine of the nutritional assessment of patients.

Based on the current research, different screening tools and nutritional parameters were compared. We confirmed that the overall prevalence of malnutrition for the elderly patients admitted to the surgery department of the Second Affiliated Hospital, Harbin Medical University, China ranged from 12 to over 71 %. The highest prevalence of malnutrition was detected by TLC and the lowest by CC.

It is noteworthy that malnutrition is consistently high in newly institutionalized elderly surgery patients. With MNA-SF, 64 patients (45 %) were evaluated as under-nourished. The NRS2002 indicated that 38 % patients were under-nourished and this condition was correlated with age (r = 0.33, *P* < 0.001). Older patients with digestive system disease were also likely to suffer from malnutrition. This difference is particularly relevant with NRS-2002, as this tool has an age adjustment feature for patients older than 70 years. Our Chinese patient malnutrition rates are different from those reported in the literature, which showed respectively 25 % and 53.6 % of elderly patients exhibited signs of malnutrition [28, 29]. With regard to the two assessment tools, BMI, Alb, TLC, Hb, and anthropometric data, values were lower in under-nourished patients.

Our results revealed that only a moderate agreement was found between the NRS2002 and MNA-SF (k = 0.5961), indicating that these nutritional assessments identify different at-risk groups. Both tools showed a significant association with traditional single nutritional indexes (anthropometric and biochemical markers, *P* < 0.05, except for TLC, *P* > 0.05). These results illustrate the differences in nutritional risk detected by different screening tools. In addition, nutritional status correlated inversely with BMI, anthropometric data, Alb, TLC and Hb. However, gender has no association with nutrition status, identified by both NRS2002 and MNA-SF.

Regarding the variables studied, albumin is commonly considered to be an insensitive marker of nutritional status. Studies have demonstrated that low serum Alb concentrations correlate with a longer hospital stay, medical complications, and increased mortality [30]. In the present study, an optimal nutritional status was associated with a higher level of serum Alb, however, prevalence of malnutrition detected by Alb is lower than MNA-SF and NRS2002. This finding shows that Alb does not precisely and sensitively assess malnutrition in our patients.

As reported in another study, malnutrition is also under diagnosed when BMI is used as the sole criteria, regardless of gender [31]. The prevalence of malnutrition diagnosed by BMI was far below that detected by MNA-SF and NRS2002. The possible reason for the under diagnosis of malnutrition by BMI is most likely related to fluid and electrolyte retention in those patients, leading to an overestimation of their measured ‘true’ weight [32].

In this paper, HGS has been proposed as a simple, quick, reliable, economical and objective measure of nutritional status [33]. The loss of body protein in under-nourished has negative implications on muscle strength and functional status [34]. The literature provides a clear understanding that HGS is useful in the evaluation of protein energy malnutrition (PEM) in conjunction with other parameters [35]. ASPEN recommended a standardized set of six characteristics for the diagnosis of under-nourished, one of which is muscle strength [36]. However, HGS has some limitations. Bin et al. noted that while the sensitivity was high, HGS had a very low specificity, which would have implications for false positive diagnoses [37]. The cut off points for the identification of patients at risk of malnutrition by weak HGS are not clear or consistent between studies [38]. In addition, our study allowed us to compare the association between HGS and other nutritional assessment parameters.

The objective of nutritional screening is to identify accurately those patients who are under-nourished and who will benefit from nutritional treatment. Better nutritional screening tools should be highly sensitive and specific [39]. In the present inquiry, NRS-2002 had a higher specificity and better positive and negative predictive values than MNA-SF, while MNA-SF showed a higher sensitivity.

Our study had a number of limitations. For example, the small sample size may limit the power of data analysis. As assessment tools, both MNA-SF and NRS2002 consist of both history taking and physical examination of the patients. Thus, during our study, since some patients could not remember their exact body weight and or the details of their dietary intake, the relevant information had to be obtained from the recall of patients and their relatives.

In conclusion, the prevalence of malnutrition in hospitalized surgery patients over 65 years old was high. Thus, the early diagnosis of patients, who are at risk from malnutrition or who are malnourished, is essential so that prompt treatment can be initiated. Although the diagnosis of nutritional status varied according to the method used, both tests correlated with each other and with age, BMI, anthropometric data and laboratory tests in hospitalized surgery patients. Therefore, Both MNA-SF and NRS2002 are simple, inexpensive, reliable, economical and objective measures for assessing the nutritional status of Chinese elderly inpatients in a surgical ward. Regardless of the screening method used, we feel that all patients should undergo an evaluation of their nutritional status upon admission to hospital and at least once more during their stay.
